# Risk of cancer after ST-segment-elevation myocardial infarction

**DOI:** 10.1007/s10654-023-00984-8

**Published:** 2023-03-22

**Authors:** Maarten J. G. Leening, Nathalie I. Bouwer, M. Arfan Ikram, Maryam Kavousi, Rikje Ruiter, Eric Boersma, Ewout-Jan van den Bos, Auke P. J. D. Weevers, Jaap W. Deckers, Mark-David Levin

**Affiliations:** 1 Department of Cardiology, Erasmus MC – University Medical Center Rotterdam, PO Box 2040, 3000 CA Rotterdam, the Netherlands; 2 Department of Epidemiology, Erasmus MC – University Medical Center Rotterdam, Rotterdam, the Netherlands; 3 Department of Cardiology, Albert Schweitzer Hospital, Dordrecht, the Netherlands; 4 Department of Internal Medicine, Albert Schweitzer Hospital, Dordrecht, the Netherlands; 5 Department of Epidemiology, Harvard T.H. Chan School of Public Health, Boston, MA USA; 6 Department of Internal Medicine, Maasstad Hospital, Rotterdam, the Netherlands

**Keywords:** ST-segment-elevation myocardial infarction (STEMI), Cancer, Paraneoplastic, Prognosis, Population-based, Epidemiology

## Abstract

Analyses from administrative databases have suggested an increased cancer incidence among individuals who experienced a myocardial infarction, especially within the first 6 months. It remains unclear to what extent this represents an underlying biological link, or can be explained by detection of pre-symptomatic cancers and shared risk factors. Cancer incidence among 1809 consecutive patients surviving hospitalization for thrombotic ST-segment-elevation myocardial infarction (STEMI; mean age 62.6 years; 26% women; 115 incident cancers) was compared to the cancer incidence among 10,052 individuals of the general population (Rotterdam Study; mean age 63.1 years; 57% women; 677 incident cancers). Pathology-confirmed cancer diagnoses were obtained through identical linkage of both cohorts with the Netherlands Cancer Registry. Cox models were used to obtain hazards ratios (HRs) adjusted for factors associated with both atherosclerosis and cancer. Over 5-year follow-up, there was no significant difference in the incidence of cancer between STEMI patients and the general population (HR 0.96, 95% CI 0.78–1.19). In the first 3 months after STEMI, cancer incidence was markedly higher among STEMI patients compared to the general population (HR 2.45, 95% CI 1.13–5.30), which gradually dissolved during follow-up (*P*-for-trend 0.004). Among STEMI patients, higher C-reactive protein, higher platelet counts, and lower hemoglobin were associated with cancer incidence during the first year after STEMI (HRs 2.93 for C-reactive protein > 10 mg/dL, 2.10 for platelet count > 300*10^9^, and 3.92 for hemoglobin < 7.5 mmol/L). Although rare, thrombotic STEMI might be a paraneoplastic manifestation of yet to be diagnosed cancer, and is hallmarked by a pro-inflammatory status and anemia.

*Trial registration* Registered into the Netherlands National Trial Register and WHO International Clinical Trials Registry Platform under shared catalogue number NTR6831.

## Introduction

Individuals diagnosed with cancer face increased short-term risks of cardiovascular events [1] which is often attributed to invasive procedures and chemotherapy. Conversely, analyses from large administrative databases have suggested an increased cancer incidence among individuals who experienced a myocardial infarction, especially during the first 6 months after hospital admission [2–4]. From these studies it remains unclear, however, to what extent this finding represents an underlying biological link, or can be explained by increased detection of pre-symptomatic cancers in a population receiving comprehensive clinical work-up (i.e. asymmetrical follow-up) and/or shared risk factors for both atherosclerosis and cancer (i.e. confounding) [2–6].

We aimed to study cancer incidence and its temporality among patients with thrombotic ST-segment-elevation myocardial infarction (STEMI). As a secondary objective, we sought to identify indicators of short-term cancer incidence among STEMI patients.

## Methods

**Fig. 1:**
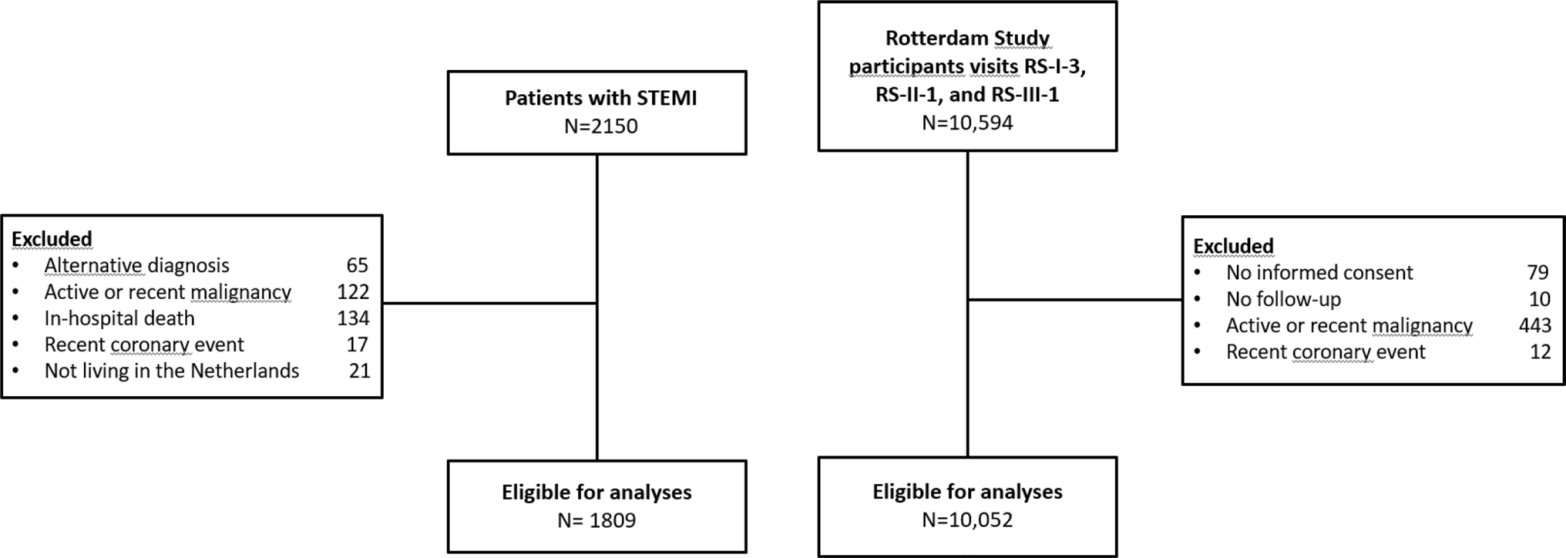
Flowchart of the study population included

We reviewed all consecutive STEMI admissions between 2010 and 2017 at a single regional primary percutaneous coronary intervention center. Patients with rare non-thrombotic causes of STEMI and patients who died during the index hospitalization were excluded, resulting in 1809 patients for analysis. Cancer incidence in these patients was compared with that of an unselected prospective population-based cohort in a neighboring city (the Rotterdam Study; visits RS-I-3, RS-II-1, and RS-III-1; n = 10,052). The objectives and design of the Rotterdam Study have been described in detail previously [7]. For both the STEMI cohort and the Rotterdam Study cohort, individuals with active malignancy, malignancy diagnosed ≤ 5 years before baseline, or coronary intervention ≤ 90 days before baseline were excluded (Fig. 1) [8, 9].

Data on pathology-confirmed cancer diagnoses (not including non-melanoma skin cancers) were obtained through identical linkage of both cohorts with the Netherlands Cancer Registry of the of the Netherlands Comprehensive Cancer Organization [9]. Follow-up started at the date of STEMI hospitalization or Rotterdam Study research center visit, and was truncated at date of pathology confirmed-cancer diagnosis, death (14.6 per 1000 person-years among the STEMI patients and 12.1 per 1000 person-years among the Rotterdam Study population), loss to follow-up (0.9% of the STEMI patients and 0.3% of the Rotterdam Study population), linkage with the cancer registry, or 5 years of follow-up (to account for longer follow-up in the Rotterdam Study), whichever came first.

Following a pre-specified analysis plan, Cox models were used to obtain hazards ratios (HRs) for incident cancer among STEMI patients compared to the Rotterdam Study population. All analyses were adjusted for factors associated with both atherosclerotic cardiovascular disease and cancer: age, sex, smoking, diabetes, body mass index, and C-reactive protein [5, 6]. Based on prior data, [2–4] we stratified the 5-year follow-up period into pre-specified time strata of 0–3, 3–6, 6–12, 12–24, and 24–60 months.

Continuous candidate risk indicators for cancer diagnosis in the first year after STEMI hospitalization were standardized for comparison of corresponding age- and sex-adjusted HRs. However, if proven significant after Bonferroni correction, adjusted HRs for clinically meaningful thresholds were additionally presented in order to enhance clinical interpretation.

IBM SPSS Statistics version 25 was used for all statistical analyses.

## Results

Baseline characteristics of the STEMI patients and Rotterdam Study general population sample are displayed in Table 1. A total of 26.0% of the STEMI patients were women, and the median age was 62.6 years. Among the Rotterdam Study participants 57.3% were women and median age was 63.1 years. Of the included STEMI patients, 1.7% presented with a resuscitated cardiac arrest. Nearly all STEMI patients (99.4%) underwent coronary angiography, of whom 95.2% underwent primary percutaneous coronary intervention and 2.5% underwent urgent coronary artery bypass grafting. Significant obstructive coronary disease in non-culprit coronary arteries was present in 38.5% of these patients.

**Table 1:** Baseline characteristics

	STEMI patients	Rotterdam study
	n = 1809	n = 10,052
Age, years *	62.6 (54.0–71.8)	63.1 (57.9–72.0)
Women	471 (26.0)	5762 (57.3)
*Smoking status*		
Current	357 (19.9)	2253 (22.7)
Former	817 (45.6)	4596 (46.3)
Never	618 (34.5)	3072 (31.0)
Body mass index, kg/m^2^ *	26.3 (24.4–29.1)	26.7 (24.3–29.5)
Total cholesterol, mmol/L	5.1 (1.2)	5.7 (1.0)
High-density lipoprotein cholesterol, mmol/L	1.2 (0.3)	1.4 (0.4)
Hypertension	901 (49.8)	3876 (39.5)
Diabetes mellitus	279 (15.4)	755 (7.5)
Aspirin use	287 (15.9)	1359 (13.5)
Anticogulant use	60 (3.3)	314 (3.1)
History of coronary heart disease:	250 (13.8)	690 (6.9)
Myocardial infarction	208 (11.5)	502 (5.0)
Percutaneous coronary intervention	174 (9.6)	190 (1.9)
Coronary artery bypass grafting	42 (2.3)	239 (2.4)
History of cerebroascular disease	117 (6.5)	675 (6.7)
History of venous thrombo-embolism	34 (1.9)	324 (3.2)
Chronic obstructive pulmonary disease	163 (9.0)	646 (6.4)
Glomerular filtration rate, mL/kg/1.73 m^2^ *	86 (72–97)	81 (71–91)
Renal dialysis	3 (0.2)	1 (0.0)
Sodium, mmol/L	138 (3)	142 (2)
History of cancer **	100 (5.5)	363 (3.6)
Hemoglobin, mmol/L	8.9 (1.0)	8.8 (0.8)
Platelet count, 10^9^/L	251 (70)	266 (62)
Leukocyte count, 10^9^/L *	10.6 (8.4–13.2)	6.6 (5.6–7.8)
C-reactive protein, mg/L *	2.0 (2.0–7.0)	1.6 (0.6–3.5)

Unimputed data. Values are counts (percentages) or means (standard deviations)

*Median (25th–75th percentile) because of skewed distribution

**Not including non-melanoma skin cancer

**Fig. 2:**
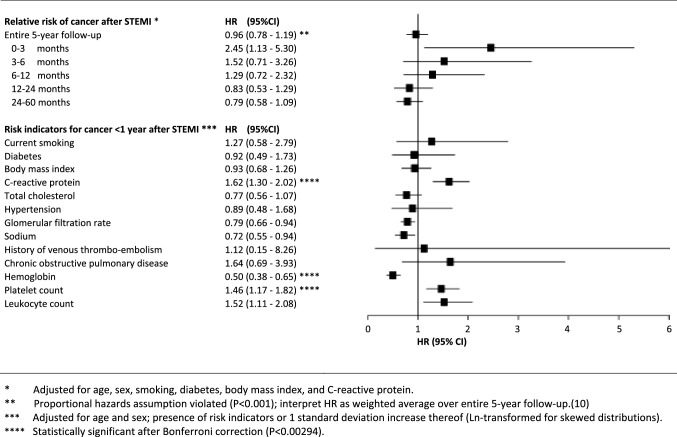
Relative risk of cancer after STEMI and risk indicators for cancer among STEMI patients

Over the entire 5-year period, 115 STEMI patients and 677 general population individuals were diagnosed with cancer (crude incidence rates 16.5 and 14.3 per 1000 person-years, respectively; Table 2). Over the full 5-year follow-up, there was no significant difference in the incidence of cancer between STEMI patients and the general population (Fig. 2; HR 0.96, 95% CI 0.78–1.19). However, the proportional hazards assumption was violated [*P* < 0.001 for Ln(follow-up time) interaction term], indicating that hazards were not stable over the 5-year follow-up period [10]. In the first 3 months after STEMI, cancer incidence was markedly higher among STEMI patients compared to the general population (HR 2.45, 95% CI 1.13–5.30). Differences in cancer incidence between STEMI patients and the general population gradually dissolved during follow-up (P-for-trend 0.004).

**Table 2:** Incident cancer diagnoses

	STEMI patients	Rotterdam study
	n = 1809	n = 10,052
All incident cancers	115	677
*Cancer subtypes*		
Upper gastrointestinal	10 (8.7)	30 (4.4)
Hepatobiliary and panreatic	2 (1.7)	27 (4.0)
Colorectal	15 (13.0)	118 (17.4)
Breast	7 (6.1)	107 (15.8)
Prostate	18 (15.7)	111 (16.4)
Genital	1 (0.9)	24 (3.5)
Urologic	11 (9.6)	63 (9.3)
Otolaryngol	3 (2.6)	20 (3.0)
Lung	23 (20.0)	81 (12.0)
Melanoma	9 (7.8)	26 (3.8)
Hematologic	10 (8.7)	37 (5.5)
Other/unknown primary origin	6 (5.2)	33 (4.9)
Metastases at cancer diagnosis	21 (18.3)	97 (14.3)
Incident cancers by follow-up time strata		
0–3 Months	15 (13.0)	22 (3.2)
3–6 Months	11 (9.6)	32 (4.7)
6–12 Months	16 (13.9)	66 (9.7)
12–24 Months	26 (22.6)	158 (23.3)
24–60 Months	47 (40.9)	399 (58.9)

Values are counts (percentages among total number of cancer cases)

Sensitivity analyses, including competing risk regression to account for non-cancer mortality, age- and sex-matched analysis, and complete-case analysis yielded similar estimates and patterns (data available upon request). No significant associations were observed with common cancer locations or the presence of disseminated disease at time of cancer diagnosis (Table 2).

Among STEMI patients, higher C-reactive protein, higher platelet counts, and lower hemoglobin were significantly associated with cancer incidence during the first year after STEMI (Fig. 2; age- and sex-adjusted HRs 2.93 for C-reactive protein > 10 mg/dL, 2.10 for platelet count > 300*10^9^, and 3.92 for hemoglobin < 7.5 mmol/L). History of coronary heart disease and presence of non-culprit coronary stenosis were not significantly associated with cancer incidence in any of the follow-up time strata.

## Discussion

During the initial period after hospitalization, STEMI patients appeared at increased risk of being diagnosed with (likely pre-existing) cancer. This might be explained by some STEMIs being of 'paraneoplastic' thrombotic origin, for instance due to a pro-thrombotic state induced by an asymptomatic undiagnosed cancer, as we postulated previously [11].

Our results are generally in line with findings from several administrative databases [2–4]. These previous studies were hampered by asymmetrical data collection among individuals with and without myocardial infarction, detection bias through clinical work-up of patients with myocardial infarction, or lack of available data on confounding shared risk factors.

In order to study the existence of paraneoplastic STEMI, we chose our reference population carefully to address several issues. First, we ensured identical follow-up using linkage to a nationwide cancer registry, hence ruling out asymmetrical data collection. Next, we aimed to reduce the likelihood of spurious associations by detecting asymptomatic cancers through routine clinical work-up in STEMI patients, since Rotterdam Study participants underwent comparable blood work and examinations. The only marked difference was the prevalence of thoracic imaging: > 90% of STEMI patients underwent chest radiography, while only 35.3% of the Rotterdam Study participants underwent non-enhanced cardiac CT as part of the study protocol. However, lung cancers accounted for only 13.5% of all diagnoses during the initial 3 months of follow-up. Next, in order to address shared etiology, we adjusted for cardiovascular risk factors that have been implicated in cancer development [5]. Nonetheless, residual confounding cannot be ruled out.

The lack of ethnic diversity in our study populations (95.8% of the Rotterdam Study is white) warrants replication in other populations. Also, absolute cancer risks are likely somewhat underestimated, since we only had data available on pathology-confirmed cases [9]. However, this was the case for both populations and hence is unlikely to have affected the presented relative risk estimates.

## Conclusion

In conclusion, although rare, thrombotic STEMI might be a paraneoplastic manifestation of yet to be diagnosed cancer, and is hallmarked by a pro-inflammatory status and anemia.
